# Online speech and communal conflict: Evidence from India

**DOI:** 10.1093/pnasnexus/pgaf149

**Published:** 2025-05-13

**Authors:** Sebastian Schutte, Daniel Karell, Ryan Barrett

**Affiliations:** Peace and Conflict Dynamics, Peace Research Institute Oslo, Hausmanns Gate 3, Oslo 0186, Norway; Department of Sociology, Yale University, 493 College Street, New Haven, CT 06511, USA; Institution for Social and Policy Studies, Yale University, 77 Prospect Street, New Haven, CT 06511, USA; Department of Sociology, Yale University, 493 College Street, New Haven, CT 06511, USA

**Keywords:** communal conflict, social media, Hindu nationalism, Koo, India

## Abstract

How does online speech affect offline attacks? While a growing literature has examined this link in right-wing violence in the West, much less is known about its importance in the religiously divided societies of the Global South. Furthermore, existing research has overwhelmingly focused on negative externalities of social media, while paying comparatively little attention to their conciliatory effects. We advance the scholarship in both of these areas by analyzing 22.4 million posts from Koo, an Indian social media network popular among India’s Hindu nationalists. We combine these data with information on attacks on religious minorities in India from 2020 through 2022. We find that the frequency of hashtags with a Hindu-chauvinist connotation are associated with increased attacks on Muslims and Christians. We also find that the frequency of hashtags alluding to the overcoming of religious divisions is associated with fewer attacks. These results survive a battery of robustness checks and supplemental tests. Additionally, the observed relationships disappear during exogenous Internet outages, consistent with the effect being driven by online speech. Importantly, since the content we study is not overtly aggressive and conveys values rather than factual claims, it does not classify as hate speech, misinformation, or disinformation. This suggests that the scholarly debate on what kinds of online speech influence offline harm has to be broadened and that censorship and fact-checking can fall short of addressing online speech’s negative consequences in religiously divided societies.

**Significance Statement**

We examine the relationship between online speech and offline harm in India. Drawing on the Koo social network, we focus on the effects of two frequently used religious references: (i) divisive hashtags praising Hindu deities and (ii) conciliatory hashtags signaling inter-group amity. We find that the former are positively associated with attacks against religious minorities, while the latter correlate negatively with incidents. Empirically, these results suggest that online speech plays a role in both instigating and preventing communal violence, and underscore that implicit online signaling, rather than explicit hate speech or disinformation, can lead to offline harm. Consequently, censorship and fact-checking will likely fall short when culturally specific value statements short of hate speech and overt calls for violence are used.

## Introduction

A large literature suggests that social media use can have various negative political consequences, including increased ideological polarization (1, 2), accelerated spread of mis- and disinformation (3–5), and increased political violence in Western countries (6–10).

Less research has examined social media’s effects in the religiously and ethnically divided societies of the Global South (11), particularly with regard to domestically developed platforms (12). Presumably, such effects would be quite dire: the spreading of rumors, invoking memories of past victimization, and exaggerating the threats posed by out-groups—all facilitated by the use of social media—can trigger violence (13–17). A high-profile case seems to validate these concerns: the United Nations identified Facebook as a “determining factor” in the 2016–2018 Rohingya genocide in Myanmar (18, 19).

Yet, despite good reasons to suspect that social media can have uniquely detrimental effects across the Global South, the evidence is far from clear. Upon closer inspection, Facebook propaganda in Myanmar was created in a coordinated fashion, thereby making it more akin to historical cases of traditional mass media being used to dehumanize target groups (15, 20). Additionally, Facebook availability in the country seems to have escalated violence only in places with already high ethnic tensions, while reducing conflict overall (21). Evidence from experimental deactiviations of Facebook during times of heightened group tensions adds to this complex picture. In Bosnia and Herzegovina, users who stayed offline during times of genocide remembrance reported *higher* disregard for out-groups than users who remained active (22), but this effect does not seem to generalize across countries (23). Additionally, research on exact communication strategies suggests that effective mobilization does not always require overt hate, but rather value systems and styles of extreme speech that are presented as having wide popular legitimacy (24).

Therefore, more research is required to understand how online speech contributes to offline harm in divided societies (25–30). To this end, we focus on India as an ideal case. Tensions between Hindus and Muslims in the country have risen and fallen over historical time-scales (31) and are currently high (32–34). Consequently, online speech frequently touches on group relations and digital access is rapidly expanding in India. Hundreds of millions of residents are online and a robust local technology sector is developing rapidly. With data obtained from a local social media platform, India affords us the unique opportunity to examine both the conflict-escalating and -mitigating effects of social media.

On the escalatory side, we analyze hashtags associated with an expression of Hindu faith “Jai Shri Ram” (Glory to Lord Ram, JSR hereafter). JSR is closely tied to a specific political incident: in 1992, a long-standing dispute over one religious site claimed by both Hindus and Muslims became infamous when Hindu activists demolished the 16th century Babri mosque, claiming that it had been built in the birthplace of the Hindu God Ram (35). After the destruction of the Babri Mosque, the traditional Hindu greeting “Ram Ram” gave way to the more divisive “Jai Shri Ram” (36). This phrase is not an overt reference to conflict and serves as a peaceful expression of religious devotion for many Indians, but since the demolition of the Babri mosque, it has become politicized and adopted by Hindu nationalists and members of the ruling Bharatiya Janata Party (BJP). Recently, JSR has been used as a battle cry in attacks on religious minorities, such as Muslims and Christians (37–41).

Of course, not all of India’s Hindus or BJP members seek communal conflict and many work for intercommunal harmony. Some social media users post expressions meant to avert conflict. One such expression is “Kabir.” Kabir refers to a 15th century Hindu saint who turned away from organized religion and embraced ideas compatible with both Hinduism and Islam. Roughly comparable to the European “Erasmus,” Kabir’s legacy extends into the present, with religious communities devoted to their commemoration and the building of bridges across communities (Kabir Panth, similar to ecumenical churches in the West) (42). The prevalence of the Kabir references affords us a rare opportunity to examine the possible conflict-mitigating effects of online speech. Thus, we analyze hashtags invoking the name of Kabir, as well as those proclaiming JSR.

We obtain data on the use of hashtags associated with JSR and Kabir from the Koo social media platform,^a^ which was a functional Indian equivalent to X/Twitter and popular among the country’s Hindu nationalists and BJP members. We relate this information to incidents of offline assaults, harassment, and other kinds of attacks against members of religious minority groups (i.e. Muslims and Christians). These event data come from the Documentation of the Oppressed^b^ (DOTO), a nonprofit Indian organization recording attacks on religious minorities.

While Koo is only one social media platform among many used in India, it is distinguished in terms of the audience it exposes to online speech. This becomes evident when considering online speech *production* and *consumption* separately. Like many other social (and traditional) media outlets in India, Koo allows for production of Hindutva rhetoric. However, it differs from most of these outlets in that it concentrates and exposes this rhetoric to an audience that is very receptive to Hindu religious references. With this audience, conciliatory and escalatory references in the online content are more likely to translate into action (8). This makes Koo relevant beyond India: platforms are increasingly aligned with political sides, such as Truth Social and X on the political right in the United States, and Bluesky on the left. Koo therefore represents a case from an increasingly relevant class of social networks.

Our results indicate that the frequency of JSR impressions on Koo is positively associated with attacks against religious minorities in the 10 states of India’s Hindi Belt during the 2020–2022 period. At the same time, the frequency of Kabir expressions is negatively associated with attacks against members of religious minority groups. These results are robust to different model specifications and estimators, a range of temporal aggregations, and supplemental tests. In addition, the relationships disappear in the presence of exogenous Internet outages. The results of this test further support the argument that speech on Koo is associated with subsequent attacks against religious minorities, and suggests that online activity, more broadly, can affect offline violence.

Generally, our findings advance our understanding of social media’s role in offline strife between groups in divided societies, particularly by showing how online speech can both foster and prevent conflict. In addition, our findings broaden the common focus on censoring or moderating explicit calls for violence, hate speech, and mis- and disinformation. While such efforts can be effective, we offer insights into circumstances where they are likely inapplicable: JSR and Kabir expressions mainly convey value statements and seemingly sincere religious sentiments rather than hate speech or purported facts.

## Measuring online speech and communal conflict

### Koo data

Koo, launched in 2020, became popular with Hindu nationalists and BJP members after BJP government ministers encouraged supporters to switch from Twitter to Koo. These leaders were upset that Twitter did not fully block the accounts of activists and journalists commenting on a farmers’ protest (43, 44). As a result, they issued official BJP endorsements of Koo on February 9, 2021, and Prime Minister Narendra Modi commented favorably on the platform in a later speech. In July 2024, the platform announced that it would shut down for financial reasons and the website is no longer accessible.

To collect data from the Koo network, we relied on an undocumented API to access profiles, posts, and metadata, following an approach first described by an Indian research team (45). Between February 13 and December 24, 2022, we resolved public Koo URLs to download 5,043,507 complete user profiles with an associated 33,647,672 public posts, shares (reposts), and comments. Due to the use of the API, no automated crawling of the site was required, keeping the traffic load on the service to a minimum. Also, by using the API, we did not violate any Terms of Service during data collection.

We estimate that the data we collected account for 80% of the social network during the study period, based on an analysis of known and unknown user IDs in the lists of followers and followees. We had intended to capture the complete network, but Koo introduced authentication keys at the end of 2022, thereby removing public access to their data. See Materials and methods, as well as Section 1 of the Supplementary material, for more information on Koo and the data collection.

Since the Koo network launched in early 2020, there is an initial period captured by our data collection that does not lend itself to meaningful analysis. During this period, Koo was creating test posts and the volume of user-generated content was low. Therefore, we left-censor our analysis to exclude this initial period and analyze data starting on September 1, 2020. Additionally, we exclude observations toward the end of the data collection period. Since the download lasted 10 months, we end our analysis with the first day of the download, February 13, 2022. Analysis of later dates would systematically omit newer posts from accounts that had been downloaded at the beginning of data collection since we visited accounts only once during data collection. We therefore right-censor to the first day of the download period. The resulting study period extends from September 1, 2020 to February 13, 2022.

**Fig. 1.**
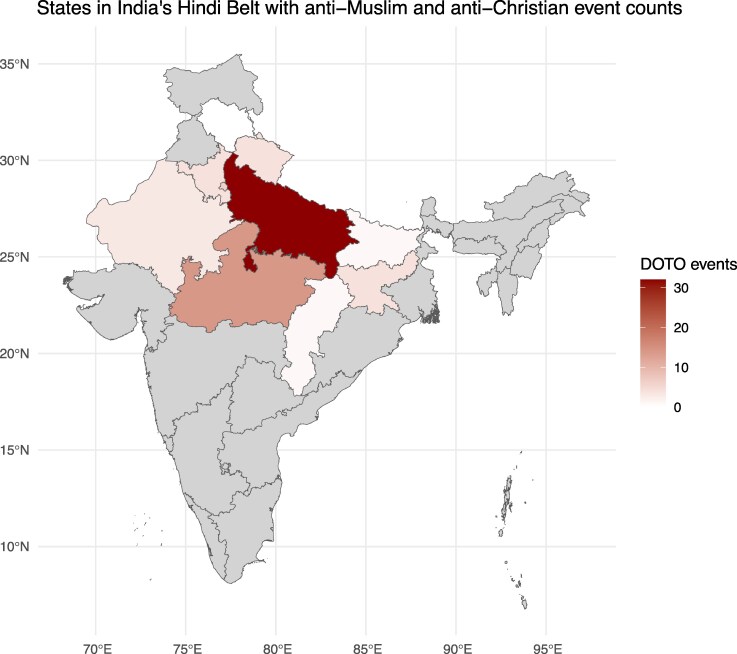
State boundaries in the “Hindi Belt” and counts of attacks against minorities in the study region and during the study period. Note the high concentrations in Uttar Pradesh, the central northern state with more than 240 million inhabitants and the site of the Babri mosque demolition.

To study the effects of social media use on communal conflict, we focus on the “Hindi belt,” ten states in central India where Hindi is the majority language and communal clashes are relatively frequent (Figure 1). To do so, we subset the Koo observations to those that the platform identifies as Hindi and we subset the DOTO data to the ten states in the Hindi Belt.

Finally, we compute daily counts of JSR and Kabir impressions. The counts comprise posts, likes, and shares that contain either the English hashtags #JaiShriRam and #Kabir, their Hindi equivalents, or hashtags using alternative spellings and synonyms of JSR and Kabir. We identify the alternative spellings and synonyms using a word embedding model (see Materials and methods and Section 4 in the Supplementary material). We normalize these counts by the daily total number of Hindi Koo impressions. The normalized counts, or fractions, are our main explanatory variables. Figure S1 in the Supplementary material shows that JSR and Kabir impressions make up a relatively consistent proportion of the total impressions across days of the week.

**Fig. 2.**
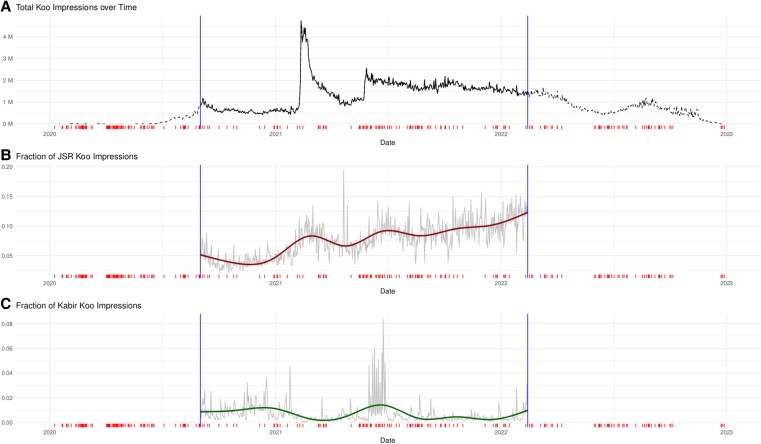
Timeline of total impressions in the Koo network in millions (A) and fractions of JSR (B) and Kabir impressions (C). The vertical blue lines demarcate the study period. The red ticks along the horizontal axes show days with attacks on religious minorities. Note the spike in early 2021 after the BJP endorsement of Koo. The decline of total Koo activity after the end of the study period is an artifact of the data collection (see Section 1 of the Supplementary material).

Figure 2 displays the total number of Koo impressions for the study period (top) and the fractions of JSR and Kabir expressions (bottom rows). The noticeable peak in overall impressions in early 2021 followed the BJP’s aforementioned official endorsement of Koo after the standoff with Twitter. The rugs displayed in the timelines are days with attacks on religious minorities.

### Attacks on religious minorities

We obtain records of attacks against religious minorities—mostly Muslims, but also some Christians—from DOTO’s public website. Each record of an attack includes information on the date, district-level location, and the religious identities of victims, as well as other information. Not all incidents involve physical violence; some consist of harassment, intimidation, and property damage. As with the Koo data, we limit our DOTO data to the Hindi Belt and the study period. Figure 1 shows the total count of attacks by state in the study region. We observe a total of 99 events in this region during the study period, and 160 events during the full download period. See Materials and methods and Sections 1, 2, and 7 in the Supplementary material for further information about the data from DOTO, as well as analyses using other event data to check the robustness of our main results.

### Panel dataset

We combine the Koo and conflict observations into a state-day panel dataset. We follow previous research in assuming that Indian states have agency in tolerating varying levels of intercommunal violence within their territories (46, p. 64ff). That is, while district-level aggregations would lead to more statistical observations, law enforcement and political responses to riots are the responsibility of the state, which makes it the appropriate unit of analysis. Beyond theoretical considerations, a district-level analysis could also artificially inflate the number of observations and run the risk of statistical overfitting (47). We merge this dataset with additional time-varying variables, such as the occurrences of Internet outages. See Materials and methods for details and Table S11 in the Supplementary material for descriptive statistics.

## Results

### Main analysis

**Fig. 3.**
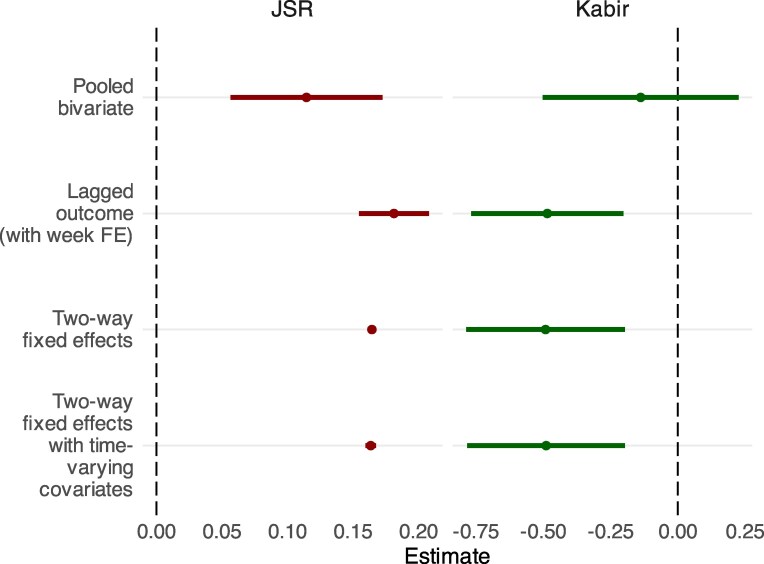
Inferential results from the main analysis. Horizontal bars denote the 95% CI.

The main analysis of the relationship between the prevalence of JSR and Kabir hashtags on Koo and attacks on religious minorities consists of four regression models using ordinary least squares.^c^ We begin with a pooled bivariate regression, in which we regress the number of attacks on the previous day’s normalized count of JSR and Kabir impressions (with standard errors clustered by state and day). As shown in the first row of Fig. 3, we find that the prevalence of JSR is positively associated with attacks while the prevalence of Kabir is negatively associated with attacks. The former relationship is significant at conventional levels (P<0.001); the latter is not. The Kabir predictor’s lack of significance could be due to a combination of the fewer number of Kabir impressions, relative to JSR impressions, and notable time heterogeneity due to the COVID-19 pandemic, which we elaborate on in the following paragraph.

Our second model adds a lagged outcome term, regressing the number of attacks on the previous day’s normalized count of JSR and Kabir impressions and that day’s number of attacks. This “lagged dependent variable” (LDV) strategy is one way to account for possible unobserved confounding since the lagged outcome term adjusts for existing factors associated with the outcome (48, 49). The model also includes week fixed effects (FEs) since the pandemic—including the “Delta” COVID variant, which was discovered and particularly deadly in India (50, 51)—occurred during our study period. During the pandemic, the Indian government attempted to slow the spread of the virus in a series of national and regional lockdowns (52). The lockdowns likely had direct effects on the opportunity to engage in attacks as many people stayed indoors and changed other social behavior (28). Modeling these shifts in behavior based on observables would be challenging as reliable data on daily mobility across the subcontinent is unavailable, so we rely on week FEs to help account for changes in the rates of COVID infections and related governmental policies. The results of this second model indicate that JSR impressions are associated with an increase in subsequent attacks (β=0.181, P<0.001) while Kabir expressions are associated with a decrease in subsequent attacks (β=−0.485; P<0.01) (Fig. 3).^d^

The third model in our main analysis regresses offline attacks on the hashtag variables with the inclusion of state and week FEs, but no other covariates (i.e. a two-way fixed effects specification, or TWFE). We include state FEs because large variation can be seen in the prevalence of attacks across states (Fig. 1), which are largely the result of long-term political and demographic processes. As seen in the third row of Fig. 3, we once again find a positive association between JSR expressions and later attacks (β=0.164; P<0.001) and a negative association between Kabir expressions and later attacks (β=−0.491; P<0.01).

The final model in our main analysis adds two time-varying controls to the TWFE specification: the number of Internet outage in a state, lagged by 1 day (corresponding to the Koo variables’ lag), and data from the “Integrated Crisis Early Warning System” (ICEWS) (53), which capture time-varying political relations between India and (Muslims-majority) Pakistan. This model provides evidence consistent with our previous models. The prevalence of JSR impressions shows positive associations with the number of subsequent attacks against religious minorities (β=0.163; P<0.001), while the prevalence of Kabir impressions is negatively associated (β=−0.489; P<0.01) (Fig. 3). This final model is our preferred specification, and the one we use in our robustness checks and additional tests.^e^ All of our models cluster standard errors at the state and day level. Table S5 in the Supplementary material presents the results in detail.^f^

### Robustness checks and additional tests

Our main results survive multiple robustness checks and supplemental tests. First, the main results are robust to using larger temporal aggregations for the lagged Koo predictors. Specifically, we use the preferred model specification to regress DOTO incidents on the mean prevalence of JSR and Kabir posts over the preceding 2 to 7 days. In other words, this check examines the possibility that social media activity takes multiple days to encourage attacks on religious minorities. When using these larger temporal aggregations, we obtain results consistent with our main results (Table S6 in the Supplementary material).

We also find similar results when using an alternative end date for the data, the last day of data collection, December 24, 2022. As earlier explained, we have incomplete records of posts for the accounts visited initially during data collection, so we include data from the entire download period only as a robustness check. Drawing on the complete dataset, we again find that associations between JSR impressions and offline events remain positive and significant at conventional levels (except when using the pooled bivariate model). The associations between Kabir impressions and the events remain negative, albeit no longer significant at conventional levels (Table S7 in the Supplementary material).

We additionally obtain results consistent with our main results when using five alternative inferential models. The first of these models adds a lagged outcome term to our preferred specification, the TWFE with time-varying covariates. The second adds an interaction of states and weeks to our preferred specification, which accounts for differential trends across spatial units over time (48). The third and fourth are a LPM and a logistic regression, respectively, both using a binary version of our outcome variable and our preferred specification. The fifth is a Poisson generalized linear model with our preferred specification. Table S8 in the Supplementary material, presents the results of these alternative models.

In addition to our robustness checks, we conduct three tests of our interpretation of the results. Recall that we interpret our empirical results as evidence that social media expressions of religious and cultural divisiveness among Hindus, and particularly Hindu nationalists, increase subsequent attacks against religious minorities in India. In addition, expressions invoking the overcoming of group boundaries decrease subsequent attacks. One way to check the temporal order of implied in our interpretation—other than lagging the variables measuring Koo expressions, which we do—is to reverse the order of the explanatory and outcome variables in our models. When we do this, we find no evidence of a statistical association (Table S9 in the Supplementary material). These results suggest that our interpretation of the relationship’s direction is correct.

Our second test is a placebo treatment test, or re-fitting the preferred model with a different predictor variable. This placebo treatment should be similar to the explanatory variables of interest—for instance, it should be of interest to most Hindu residents of the Hindi Belt and have the potential to elicit emotions and reactions—but be unlikely to cause the outcome. It should also vary temporally during study period, like our Koo explanatory variables. If we found that the placebo treatment statistically predicted the outcome, this would be evidence for a flawed research design or for unsupported conclusions (61). In our test, we use the normalized count of a hashtag referring to a popular sport in India, cricket (i.e. #cricket and related hashtags), as the predictor. We find no evidence of a relationship (Table S9 in the Supplementary material), which adds further support for our interpretation of the main results.

The third test leverages the BJP endorsement of Koo. If certain expressions encourage or discourage attacks against religious minorities, then likely perpetrators of the attacks—Hindu nationalists and BJP members—must be exposed to these particular posts. In other words, this test underscores the importance of speech consumption: Koo’s ability to concentrate and expose online speech to a particular audience of likely perpetrators helps explain how it can play a role in increasing or decreasing rates of offline attacks against religious minorities.

**Fig. 4.**
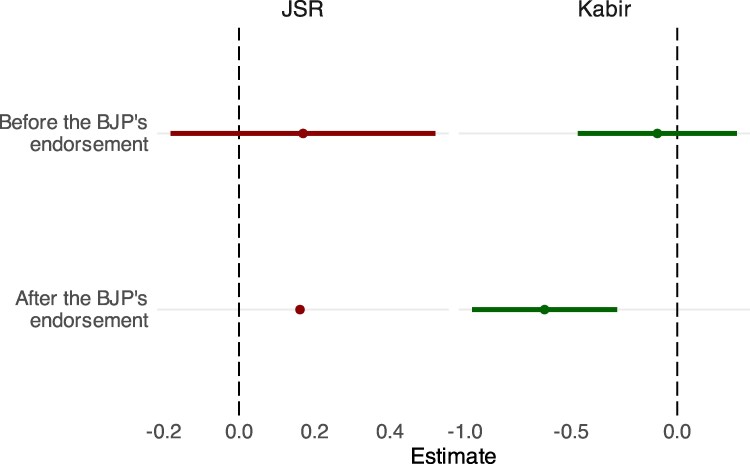
The relationship between JSR and Kabir impressions and subsequent offline events exists only after the BJP’s endorsement of Koo. Bars denote 95% CI.

We can test this logic underlying our interpretation using the BJP’s official endorsement of the platform. Since fewer Hindu nationalists and BJP followers used Koo before the endorsement on February 9, 2021 than after the endorsement (see the discussion of Fig. 2), the relationship between JSR or Kabir impressions and subsequent attacks should exist only after the endorsement, or when likely perpetrators are consuming the JSR and Kabir posts. Figure 4 shows that this is the case. We see no statistically significant relationship between JSR or Kabir and subsequent offline attacks before the endorsement, whereas we do find evidence of the relationships after the endorsement. See Table S10 in the Supplementary material, for detailed results.

While our main analysis, robustness checks, and additional tests provide compelling evidence that certain speech on Koo affects offline intercommunal strife, it remains possible that the relationship we find is spurious. For example, an overall mood in the country could lead to an increase in both JSR expressions and attacks on Muslims. Furthermore, the possibility of spuriousness also threatens our argument that *online* speech matters. It could be, for example, that discourse in print media drives both JSR expressions and attacks on minorities.

To investigate this possibility, as well as obtain further evidence supporting the importance of social media activity, we conduct a final test using data on district-level Internet outages. Specifically, we interact the JSR and Koo explanatory variables with the number of a state’s districts affected by Internet outages (both lagged by a day). If the online prevalence of JSR and Kabir on Koo is affecting offline conflict—and if online speech does in fact matter—then we should observe the previously detected relationship between the online expressions and attacks break down as Internet outages increase.^g^

**Fig. 5.**
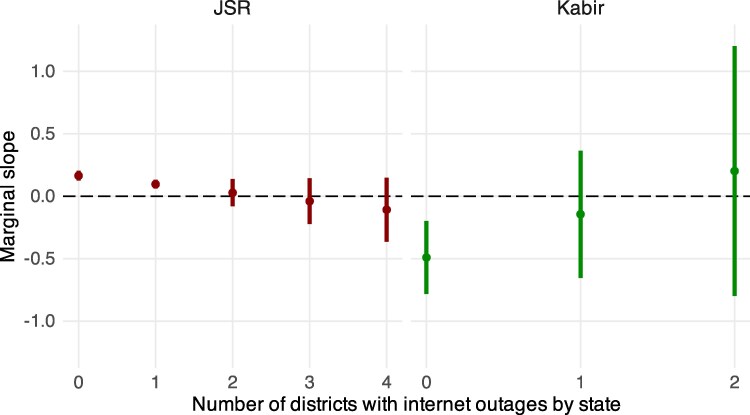
The relationship between JSR and Kabir impressions and subsequent offline events depends on Internet availability. Ribbons denote 95% CI.

Figure 5 shows that when there are no reported Internet outages, our main finding remains. However, when the number of outages increase, there is no relationship between the activity on Koo and offline attacks. We find consistent results when we dichotomize our measure of Internet outages and examine whether any Internet outage in a state nullifies the relationship between the online expressions and offline incidents (Fig. S2 in the Supplementary material).^h^ The results of this final test further support our interpretation of the empirical findings and provide new evidence that the effects of JSR and Kabir impressions do depend on social media activity.

## Discussion

This study advances our understanding of online speech and offline harm in important ways. Previous scholarship has overwhelmingly researched this link in Western settings rather than the religiously divided societies of the Global South. In addition, they have focused on a small set of Western-developed platforms (12), which, while used across the world, may have different effects from domestically or regionally developed platforms. To extend the scope of existing scholarship, we have incorporated data on millions of social media posts from an Indian platform, communal attacks, and Internet outages. By combining these data sources, we have found that both conflict-escalating and conflict-mitigating hashtags affect communal attacks in India.

Our findings underscore how platforms such as Koo can produce certain speech and facilitate its consumption by a particular audience, whose behavior can alter rates of violence. Such concentrations of receptive target audiences can lead to more efficient coordination and more easily shifted perceptions of what their peers and community think is acceptable (8), whether it be enacting or forgoing violence. Such messaging could have an outsized impact among Hindu nationalists, as they continuously produce narratives that reactivate memories for their audiences, which has been characterized as a dramatic production involving cycles of calm and tension (13).

Beyond its geographic scope, our analysis suggests that the debate about *how* online speech affects offline harm needs to be broadened. Public discourse in India and journalistic accounts have long stressed the increasingly incendiary nature of JSR expressions, and their role in signaling attacks on religious minorities (37–41).

Yet, most JSR expressions on Koo are embedded in positive messaging and appeal to common identity rather than out-group defamation. They generally do not classify as hate speech (see Section 3 and Table S3 in the Supplementary material). Moreover, religious value statements by their very nature cannot be factually incorrect. While the majority of research on social media and violence sees proximate causes in hate-speech and mis- and disinformation, we see evidence for JSR falling into a category of speech which is not overtly violent, but powerfully evocative of group-specific memories and sentiments (62–64). For example, JSR expressions can trigger hostile emotions associated with the Babri Mosque, which was built under temporary Muslim rule centuries ago, and was eventually destroyed in 1992. The expression is also tied to the subsequent construction of a temple devoted to Ram in the same place and, by triggering the emotions associated with this history, to attacks against non-Hindus today.

Finally, we also find that such cultural references cut both ways: appeals to peaceful coexistence in form of Kabir expressions are associated with reductions in communal attacks. Such positive externalities are an often overlooked aspect in the literature and warrant further attention (12, 22, 23).

As a whole, our study’s empirical insights have implications for social media governance (65). If real-world harm was simply tied to hate speech and mis- and disinformation, censorship and fact-checking could solve the problem. However, for the problem we describe, these measures seem inadequate and a broader, culturally specific debate seems warranted (66).

These conclusions rest on a data analysis including comprehensive robustness checks and supplemental analyses and tests. Yet, there are limitations of the research design that warrant further investigation. Some of these regard the analyses enabled by our data. For example, joining the various data sources was only possible at the state level. Alternative, yet similar, data sources could be used for lower levels of aggregation. However, we used the highest quality data of which we were aware. In addition, future research could also leverage more of the content of social media posts. In our case, though, the posts in our sample are only 134 characters long on average (including emojis). At this brevity, natural language expressions across multiple languages and cultural references are more challenging to classify in a meaningful and accurate way than only hashtags. A restricted focus on a smaller portion of verbose and overtly political content would likely be necessary for such a design. Furthermore, we cannot examine whether specific groups or accounts drive overall engagement with JSR and Kabir posts. Doing so would require attempts to code political or social affiliations for the obtained user accounts, based on identifying information, which would exceed the scope of the study’s data protection impact assessment. However, previous research suggests that cultural references made by some carry more weight than the same references made by others (63).

Other limitations relate to our research design. Observational studies such as ours cannot deliver proof of causal effects. Instead, our main analyses and additional tests provide results that are consistent with JSR and Kabir expressions causing variation in attacks. Finally, the differences between Koo and large social media platforms, such as Facebook and Instagram, motivate further investigation into how our insights do or do not apply to widely used, long established platforms. However, Koo shares some important similarities with a growing kind of social media platform: smaller platforms used primarily by relatively cohesive ideological- or identity-based communities, such as Truth Social and X among conservatives in the United States and Bluesky among liberals. Like these platforms, Koo delivers specific types of content to receptive communities (8). Future research could build on our study, potentially by adopting a comparative design, to shed light on the broader consequences of an increasingly ideologically fractured social media landscape.

## Materials and methods

### Obtaining and preparing the Koo data

With a previously published list of 4.1 million Koo account IDs (45), we used an undocumented REST API to resolve the accounts’ public URLs and then download their information.^i^ Developer keys or other forms of authentication were not required at the time, and Terms of Service were only in place for users from India and the United States. This yielded 3.95 million accounts that were still active during the first data collection period, February 13 through September 19, 2022. By searching these accounts’ follower lists, we identified 2.4 million new accounts and then downloaded 1.1 million of these starting September 27. We intended to download more, but access to the API was restricted on December 24, 2022 by the introduction of authentication keys. Before the restriction was put in place, we downloaded the accounts’ public posts, comments, likes, and shares. We obtained roughly 80% of all the accounts we know of from previous research and our own analysis of follower and followee lists. All aspects of our data handling dealt with publicly and legally available data, and internal handling of the data we approved in a detailed Data Protection Impact Assessment (Sikt reference #289287) by the Norwegian Center for Research Data, which is centrally tasked with upholding European GDPR laws. See Section 1 of the Supplementary material for more details.

### Identifying alternative spellings and synonyms of Koo hashtags

We used a word embedding approach to identify all relevant hashtag expressions of JSR and Kabir, i.e. alternative spellings and synonyms of #JaiShriRam and #Kabir. This was a critical step because an initial glance at the Koo content made it quickly clear that nonsystematic spellings and variations were common. For example, the hashtag “#jaishr**i**ram” has an alternative spelling, “#jaishr**ee**ram.” Similarly, as hashtags do not allow for blank spaces, the Hindi variants of the hashtags might separate each word in the phrase by one or two underscore characters.

Since there is no dictionary listing all spellings and synonyms of our hashtags of interest, we adopted a data-driven approach, specifically a word embedding technique. This technique represents tokens from a vocabulary, such as hashtags, as vectors of real numbers. The vectors themselves locate the tokens in a high-dimensional space that has been learned by the embedding model based on the semantic and syntactic relationships in the vocabulary. As a result, the similarity of vectors—potentially measured in various ways, such as cosine similarity—correspond to tokens’ semantic similarity. In other words, closely related and likely interchangeable tokens, such as common spelling variants and synonyms, tend to have vectors that are close to one another (67).

To construct our embedding model, we first selected a random sample of 856,483 posts (which contain text in either Hindi or English or both), comprising 19,427,047 terms (749,424 unique terms). Then, we cleaned the posts by removing emojis, URLs, an nonunicode characters, while retaining hashtags. We also removed terms that appeared <5 times in the dataset. Finally, we tokenized the posts and, using a context window of three terms, trained a word2vec 300-dimension embedding model. The training was implemented with Python’s gensim (version 4.2.0).

We used our model to calculate the top 10 nearest neighbors of #JaiShriRam and #Kabir. These neighbors can be interpreted as the terms that have the most similar meaning to #JaiShriRam and #Kabir, based on Koo users’ use of language. We then included or excluded hashtags from this list based on whether they align conceptually with JSR and Kabir (see Section 4 and Table S4 in the Supplementary material for details). To construct the main explanatory variables, we summed the daily counts of the selected hashtags and normalized them by the daily total Koo impressions.

We accomplish two goals by selecting hashtags using the embedding approach and subsequent manual inspection. First, we remove “researcher degrees of freedom” that would come with a fully manual search and selection. These could limit the credibility of the analysis, as a hypothetical back-and-forth between hashtag selection and inferential analysis would be possible. Second, we avoid a fully automated selection of nearest neighbors in the embedding space. While such a selection would possibly be predictive of events, it would not easily lend itself to substantive interpretation.

### Conflict event data

We obtained records of attacks on religious minorities from DOTO. This database is not as well known as other conflict event databases, such as GED, ACLED, and SCAD (68–70). However, we found that these more established databases do not provide the coverage required for our analysis (see Section 6 in the Supplementary material for a detailed discussion and Section 7 and Fig. S3 for a robustness test using the ACLED data collection). Data on interpersonal attacks, mob violence, and riots are very hard to obtain, especially if they only involve few victims. Heavy-tailed distributions in the frequency and severity of attacks across domains of political violence imply that many small-scale incidents occur, but go frequently unreported in international media (71, 72). Additionally, identities of victims are not systematically recorded in the available academic data collections, which limits their adequacy for our specific application.

The DOTO database circumvents these problems somewhat by being locally based and thus able to effectively collect accounts of incidents from victims and local media reports. We collected data from the database by downloading all sub-pages on the website and extracting relevant information. See Section 1 in the Supplementary material for elaboration.

DOTO’s data comprises observations of communal attacks, mostly committed by Hindus against members of other religious groups. The observations cover a wide range of attack types, most of which classify as physical violence. However, hate speech and harassment are also reported. In Section 2 of the Supplementary material, we discuss the DOTO data in more detail; Table S2 reports all observed event types in DOTO for the study region and period. Sections 6 and 7 in the Supplementary material discuss alternative event data sources and in depth and present replication results.

### Other data

The panel dataset we use in our analyses is built around the Koo and DOTO data. However, it also contains two other time-varying variables which we include in parts of the analysis. One of these variables captures daily Internet outages. This information comes from accessnow.org, a civil society organization that aggregates reports of Internet disruptions. India holds the global record for government-mandated Internet shutdowns (73), some of which are justified under the pretext of diffusing communal tensions. As earlier mentioned, we exclude these kinds of shutdowns when using these data to avoid endogeneity in our models. For further discussion, see Section 1 of the Supplementary material; Table S1 presents a list of outage justifications during the study period.

The second time-varying variable captures international dynamics that could affect Hindu–Muslim relations in India. The conflict over Kashmir with neighboring Pakistan has been demonstrated to affect domestic politics (74). We therefore obtain a bottom-line estimate for diplomatic and military relations with Pakistan from the ICEWS data collection (53). ICEWS, which features more recent updates than comparable datasets, codes events on the “WEIS scale,” a single variable expressing the severity of events for comparative analysis. Negative scores are indicative of hostile interactions, whereas positive ones are related to cooperative events. One a weekly basis, we average WEIS scores for all political events between Indian and Pakistan.

## Supplementary Material

pgaf149_Supplementary_Data

## Data Availability

Replication data and code will be available at https://github.com/prio-data/odas_nexus25_replication.

## Notes

a See https://web.archive.org/web/20241214042109/https://www.kooapp.com/, last retrieved December 7, 2023. In July 2024, Koo announced its plans to shutdown the platform, citing financial considerations.

b See https://web.archive.org/web/20230316153253/https://dotodatabase.com/, last retrieved May 28, 2024.

c We also fit alternative models, such as a linear probability model (LPM) and a Poisson generalized linear model. We discuss these in the Materials and methods section.

d We obtain consistent results for the JSR predictor when using an LDV model without week FEs. See Table S5 in the Supplementary material.

e While this model does not explain more variance than the preceding model (see Table S5 in the Supplementary material), we designate this model as our preferred model because it explicitly adjusts for time-varying covariates that many readers would consider potential confounders.

f We note that the TWFE approach has recently generated some concern because of the risk of having negative weights and bias resulting from treatment effect heterogeneity (54–58). However, because of the “bracketing relationship” between LDV and TWFE models, which can provide the upper and lower bounds of true expected effects (48, 49, 59, 60), we see merit in our second, third, and fourth models.

g To bolster confidence in the exogeneity of the Internet outages, we do not use observations of outages attributed to “incendiary content or promoting violence,” “precautionary measure,” and “public safety/quell unrest/restore public order.” See Section 1 in the Supplementary material for discussion.

h The findings of this test are supported by some evidence that Internet outages are associated with a decrease in Koo impressions overall (β=−128.618, SE=1,592.489). (The relatively large variance, and resulting standard error, is expected given that the Koo impressions are measured countrywide whereas the outages are measured at the level of districts.) In addition, attacks on minorities do not predict Internet outages (β=−0.01, SE=0.02), which is additional evidence that the outages are exogenous (see the preceding footnote). Moreover, given that most of DOTO’s observations events are coded from local news sources that operate throughout outages, we do not believe that this effect is a mere function of reporting bias.

i This undocumented API did not grant access to all stored user information on Koo. For instance, location information of users was not revealed, although it had been previously accessible (45).

## Previous Presentation

These results were previously presented at the annual meeting of the European Political Science Association in Glasgow, 2023.
