# Supplementary material for: Online speech and communal conflict: Evidence from India
Source: PNAS Nexus. 2025 May 13;4(5):pgaf149. doi: 10.1093/pnasnexus/pgaf149 (PMC12103975; doi:10.1093/pnasnexus/pgaf149)
Supplement: pgaf149_Supplementary_Data [file pgaf149_supplementary_data.pdf]

Supporting Information for

Online Speech and Communal Conflict: Evidence from India

Sebastian Schutte, Daniel Karell, and Ryan Barrett

Corresponding Author: Sebastian Schutte.
E-mail: sebastian@prio.org

This PDF file includes:

Figs. S1 to S3
Tables S1 to S11
SI References

Contents

1 Data download and replication 3

2 DOTO event types 5

3 JSR Koo contents 6

4 Data-driven selection of relevant hashtags 6

5 Results from the main analysis, robustness checks, and additional tests 10

6 Discussion of alternative event data sources 19

7 Replication of the analysis with ACLED event data 19

List of Figures

S1 JSR and Kabir posts by week day . . . 17
S2 Moderation results (binary specification) . . . 18
S3 Main results when using ACLED . . . 20

List of Tables

S1 Government justifications for outages . . . 5
S2 DOTO event categories for study region and period . . . 7
S3 Evoked emotions by JSR posts . . . 8
S4 Selection of JSR and Kabir variants . . . 9
S5 Results of the main models . . . 10
S6 Results of the preferred model using the Koo predictors with larger temporal aggregations 11
S7 Results of the main models when using the late end date . . . 12
S8 Results of the alternative models . . . 13
S9 Results of additional tests . . . 14
S10 Results of the endorsement test . . . 15
S11 Descriptive statistics for the panel data . . . 16

1. Data download and replication

Koo. To collect data from Koo, we relied on a published list of 4.1 million Koo account IDs and the description of an undocumented API (1). We used this REST API to resolve public URLs, consisting of user IDs and fixed elements, such as https://www.kooapp.com/apiV1/users/<key>/followers. Resolving such URLs at the time led directly to raw data being delivered in JSON format.

We began data collection by downloading metadata for the known Koo account IDs. These data yielded counts for the numbers of Koos (*i.e.*, posts) made by the user and the user's numbers of followers and followees. Because these numbers were known, we could request the exactly right number of posts and followers from the API, instead of having to rely on a trial-and-error approach to find the numbers of available posts and followers. This greatly eased the computational burden on the service. Importantly, developer keys or other forms of authentication were not required at the time, and Terms of Service (ToS) were not in place for users from Europe.* The ToS in place for India and the United States did not specify the use of the undocumented API, and we did not engage in any activities forbidden for these countries.

One important change had occurred since the data had been download by a previous research team (1): the service had been moved to content delivery network (Cloudflare), which offers more redundancy than a single data center, and also enforces limits for the numbers of API requests that can be made per source IP. This is presumably a protection against Denial-of-Service attacks, which we of course fully respected. We therefore artificially delayed API requests to remain under the Cloudflare rate limit. This procedure yielded 3.95 million accounts that were still active during the first data collection period, February 13 through September 19, 2022. By searching these accounts' follower lists, we identified 2.4 million new accounts and then downloaded 1.1 million of these starting September 27. We intended to download more, but access to the API was restricted on December 24, 2022 with the introduction of authentication keys. Before the restriction was put in place, we downloaded the accounts' public posts, comments, likes, and shares. We have obtained roughly 80% of all the accounts we know of from previous research and our own analysis of follower and followee lists.

One particularity of the data collection procedure is that posts from different accounts were downloaded at different times. Since we did not revisit accounts during the download effort, observations of posts associated with the first accounts cease toward the beginning of our download period, whereas posts associated with the latter accounts were accessed later. We therefore used the first date of download as a cutoff of the data for the main analysis (although we used the entire data collection for a robustness check analysis). In addition, this approach to data downloading generates the erroneous impression that Koo network activity goes down to zero toward the end of the download period (see Figure 2 of the main text). This is simply an artifact of the data collection approach.

One legal issue needed to be navigated: the handling of personal data in Europe is governed by the GDPR law. To comply with the law, we cooperated with sikt.no.

Based on a full data protection impact assessment (Sikt reference #289287), we identified a workable solution. To ensure maximal privacy of the data, we refrained from involving any cloud services in the download. Instead, two Rasperry Pi single board computers under physical control of the research team were used for the ten-months download. Additionally, we have agreed to not share the raw data publicly.

All downloaded data are represented in a PostgreSQL database hosted in-house at the Peace Research Institute Oslo (PRIO). The import of the raw JSONs was laborious, because Koo supports

* see https://web.archive.org/web/20221118163800/https://info.kooapp.com/terms-of-service/, last accessed December 7, 2023.

all Indian languages and additionally special characters like emojis. These are not always represented in UTF-8 character encoding, and several filter steps were required to import the raw data into the database. These steps were necessary, however, as the relational database enables computationally efficient analysis of tens of millions of posts.

In summary, all aspects of our data handling dealt with public and legally available data, and its internal handling ensures maximal privacy and full adherence to the European GDPR law. Aggregated replication data and code (*i.e.*, the panel dataset) will be shared at <URL>. Scientific replication of the whole data import and analysis is possible, but this would have to be negotiated with sitk.no on a case-by-case basis, presumably involving an NDA. If readers would like to request the raw data, please contact the corresponding author.

DOTO. The "Documentation of the Oppressed" (DOTO) database was a non-profit documentation center based in New Delhi, India. Established to record verifiable incidents of hate crimes against religious minorities starting from 2014, DOTO compiled its information primarily from English and Urdu media sources (both online and print) as well as fact-finding and civil society reports.

The database focused on creating a comprehensive record of violence only against religious minorities, providing valuable insights and data for researchers and activists. It did not include attacks against other groups such as Scheduled Castes, Scheduled Tribes, Other Backward Classes for their caste identity, or targeted violence against women or LGBT communities for their gender identities. For the purposes of our study, DOTO provided unique and critical information, as conventional event data sets provide less coverage of violence only against religious minorities. (For elaboration, see the discussion in the main text's *Methods and Materials* section.) The website is currently offline, but still accessible via Internet archive.[†]

DOTO featured a simple website structure. Namely, a landing page showed a dashboard with event counts by state, victim identity, and other variables. Each described case had a dedicated sub-page with an event summary, and additional variables such as the timing, location, type of event, information on whether legal proceedings were initiated, and the reporting channel. This structure allowed for collecting data automatically in a single pass: sub-page links were collected from the landing page, and the sub-pages were then parsed to extract the detailed information. This yielded an event dataset with 1,317 events. As the data used to be public, we can share it upon request.

Internet outages. Creating the Internet outage dataset involved manually expanding the existing Shutdown Tracker Optimization Project (STOP) data set for the years 2016-2022.[‡] The data are available as a spreadsheet.[§] The STOP methodology defines an Internet shutdown as a deliberate disruption of Internet or electronic communications, making them inaccessible or unusable for a specific population or within a particular location. This definition, established in 2016 with contributions from technologists, policymakers, and activists, includes complete network shutdowns, bandwidth throttling, and service-based blocking of communication platforms. Uncertain cases are categorized separately until their nature as intentional or not is confirmed. The tracker covers global shutdowns by both government and non-state actors, indicating the perpetrator's identity when possible. In cases of non-government shutdowns, the country affected is noted, with details of the actual perpetrator listed in specific columns.

Each shutdown instance in the STOP data refers to a disruption event lasting over an hour or a

[†] see https://web.archive.org/web/20230102135253/http://dotodatabase.com/, last accessed May 28, 2024

[‡] see https://www.accessnow.org/press-release/keepiton-internet-shutdowns-2022-india/, last accessed December 7, 2023.

[§] see https://docs.google.com/spreadsheets/d/1DvPAuHNLp5BXGb0nnZDGNoiIwEeu2ogdXEIDvT4Hyfk/edit#gid=798303217, last accessed December 7, 2023. After download, we named the CSV file "keepiton.csv" and obtained an identifying sha256 hash sum of "346303473b7ecfeda050cd7a400869d5b5f5d72d8843addfa8856e31b8cbbdac".

series of related events with the same context, reasons, methods, and perpetrators. These instances are counted as single occurrences even if the Internet services are restored and shut down again, or if the scope changes. The data on shutdowns is collected from various sources, including news reports, local actors, and direct input from telecommunications and Internet companies. If a shutdown happens without a specific triggering event or as part of a broader political struggle, each is recorded as a separate instance once service resumes for at least 24 hours before any further disruptions.

Variations in shutdown counts may exist between different trackers due to methodological differences and updates. The STOP tracker does not include unconfirmed shutdowns to maintain accuracy and excludes disruptions caused by natural disasters or technical issues. For shutdowns following a "curfew" style pattern with multiple instances over time, STOP treats each as separate unless technical data confirms a continuous pattern from the same cause. This approach ensures accurate tracking and analysis of Internet shutdowns globally.

For our application, we only retained data from India. In expanding the STOP data, which consists of 1,978 Internet shutdown observations, several key changes were made. The "state" and "district" information was extracted from the "area_name_string," using automated district name matching with the `fuzzyjoin` package in `R`. We manually verified this against GADM level 2 administrative names, especially for Indian data, and cross-checked with additional information from URL sources in the original data set. This process helped identify additional districts, which were then included in the "districts" column. The data from different years was merged into a single time series spanning 2016 to 2022, ensuring consistency across columns. Finally, the duration of each shutdown was calculated using the `lubridate` package in `R`, providing deeper insight into the extent of these disruptions. The data were then matched to the main panel dataset. For the Hindi belt and the study period, 89 shutdowns are recorded in the outage data collection. Table 1 shows associated counts by government justification.

Table S1. Descriptions of the government justifications given for the Internet outages which occurred in the study region and period.

Government justification	Count
Precautionary measure	8
Public safety / Quell unrest / Restore public order	35
School exams	39
Unknown	7

For the main analysis, we did not use the complete data collection for the shutdowns. This was intended to alleviate concerns over endogeneity. Specifically, we used outages with the justifications of "School exams" and "Unknown", which are orthogonal to communal violence. This entailed removing all observations falling in the Table 1 category of "Public safety/ Quell unrest / Restore public order", comprising the specific justification of: "Fake News/ Hate Speech"; "Fake News / Hate Speech / Incendiary Content or Promoting Violence"; "Fake news / Hate speech/ Incendiary content"; "Promoting violence"; "Precautionary measure"; "Public Safety"; and "Public safety / Quell unrest / Restore public order". As a robustness check, we reran the analysis with the full set of outages and arrived at substantively identical results.

2. DOTO event types

Table S2 on page 7 shows all the different event types observed in the DOTO data collection for the study region and period. Note that many events are double-coded, for instance as "Harass-

ment/Physical assault". This is because each event can involve multiple victims and perpetrators. We report all combinations of event types as found in the data with associated counts. Additionally, we coded whether the reported events qualify as physical violence. For the study region and period, 60 events involving violence are reported and 39 non-violent events.

3. JSR Koo contents

As explained in the main text, JSR and Kabir posts appear to affect levels of offline attacks without conveying explicit hate speech. This assertion is based in our in-depth work with the posts. Due to the specifics of the "data protection impact assessment", we are not in a position to share JSR posts in full, because they could be connected back to individuals who made those posts.

As a simple remedy, we asked a native Hindi-speaking graduate student and Indian national to code a random selection of 82 posts according to the emotion expressed in the post. The full coding instructions were:

Please act as a social science researcher and help me classify a number of social media posts. The following social media posts might be in Hindi or English. Each one could be completely apolitical or even nonsensical. Many of them will have references Hindu religion in them, such as the God Ram. If this is all there is to it, then the post is not relevant for my purposes. However, if the post evokes emotions in the reader beyond that, such as anger, fear, compassion, love, or patriotism, I consider it relevant. I'm especially interested in posts that cast a specific light on religious groups, such as Hindus, Muslims, or Christians.

The first number is the post ID, followed by a semicolon. [Irrelevant formatting instructions] follow that with the primary emotion triggered in your assessment. If no specific emotion is triggered, add 'unclear'. This must be followed with another semicolon. Then, provide an explanation of your reasoning.

We intentionally left this coding instruction very open rather than suggesting pre-defined categories which could have been a poor fit for the local context. This lead to the results displayed in Table S3 on page 8.

While the number of observations here is low, the key insight is that a wide variety of emotions are expressed under the hashtag, with only a small fraction of them qualifying as hateful to observers capable of understanding context-specific cues. This provides support for our interpretation that JSR as an expression does not qualify as hate speech, even though its use on Koo is predictive of violence against religious minorities.

4. Data-driven selection of relevant hashtags

As we detail in the main text (see *Methods and Materials*), we use a word embedding model to identify alternative spellings for references to JSR and Kabir. In brief, we include nearest neighbors to "Jai Shri Ram" and "Kabir" in embedding space, with manual verification.

Table S4 on page 9 shows the exact coding choices for the top-10 English and Hindi neighbors to JSR and Kabir with explanations for the their inclusion or exclusion. The daily counts of JSR and Kabir impressions used in the main analysis are based on the included terms and associated likes and shares.

Table S2. List of DOTO events with associated counts and indication of physical violence

Event	Count	Physical Violence
Murder/Lynch	9	Yes
Physical assault	8	Yes
Harassment	7	No
HarassmentPhysical assaultThreat	5	Yes
Communal tension/Violence/Riot	3	Yes
Economic boycott	3	No
Hate Speech / Slander	3	No
Hate Speech / SlanderThreat	3	No
Physical assaultThreatVerbal Abuse	3	Yes
Attack on religious festivals/place of worshipDemolition/Attack on propertyVandalism/Hooliganism	2	Yes
Harassment/Physical assault	2	Yes
Harassment/Physical assault/Verbal Abuse	2	Yes
Harassment/Threat	2	No
Harassment/Threat/Vandalism/Hooliganism	2	Yes
Harassment/Verbal Abuse	2	No
Hate Speech / Slander/Verbal Abuse	2	No
Murder/Lynch/Physical assault	2	Yes
Threat	2	No
Attack on religious festivals/place of worship	1	No
Attack on religious festivals/place of worship/Communal tension/Violence/Riot	1	Yes
Attack on religious festivals/place of worship/Communal tension/Violence/RiotDemolition/Attack on propertyVandalism/Hooliganism	1	Yes
Attack on religious festivals/place of worship/Demolition/Attack on property	1	Yes
Attack on religious festivals/place of worship/Harassment/Physical assault/ Vandalism/Hooliganism	1	Yes
Attack on religious festivals/place of worship/Harassment/Threat	1	No
Attack on religious festivals/place of worship/Harassment/Threat/Vandalism/Hooliganism	1	Yes
Attack on religious festivals/place of worship/Threat	1	No
Attack on religious festivals/place of worship/Vandalism/Hooliganism	1	Yes
Breaking vehicles/Communal tension/Violence/Riot/Demolition/Attack on property	1	Yes
Communal tension/Violence/Riot/Vandalism/Hooliganism	1	Yes
Demolition/Attack on property/Harassment/Vandalism/Hooliganism	1	Yes
Denial of access to public spaces/institutions/Extra Legal Acquisition/appropriation of land	1	No
Denial of access to public spaces/institutions/Harassment/Physical assault	1	Yes
Denial of access to public spaces/institutions/Institutional discrimination	1	No
Economic boycott/Encounter killing/Attack/Harassment/Physical assault/Vandalism/Hooliganism	1	Yes
Economic boycott/Physical assault/Threat	1	Yes
Economic boycott/Physical assault/Vandalism/Hooliganism	1	Yes
Economic boycott/Social boycott	1	No
Economic boycott/Threat	1	No
Encounter killing/Attack/Harassment/Housing discrimination	1	Yes
Forced conversion/Harassment/Threat	1	No
Harassment/Hate Speech / Slander	1	No
Harassment/Hate Speech / Slander/Verbal Abuse	1	No
Harassment/Institutional discrimination	1	No
Harassment/Institutional discrimination/Physical assault	1	Yes
Harassment/Institutional discrimination/Unlawful Detention	1	No
Harassment/Physical assault/Sexual Harassment/Threat	1	Yes
Harassment/Physical assault/Threat/Verbal Abuse	1	Yes
Harassment/Sexual Harassment/Threat	1	Yes
Harassment/Threat/Verbal Abuse	1	No
Physical assault/Threat	1	Yes
Physical assault/Verbal Abuse	1	Yes
Rape	1	Yes
Social boycott/Threat/Verbal Abuse	1	No
Threat/Verbal Abuse	1	No
Vandalism/Hooliganism	1	Yes

Table S3. Emotions evoked by a random selection of JSR posts, according to a research assistant who is also a native Hindi-speaking Indian national.

	Evoked emotion	Freq.
1	piety	31
2	pride	9
3	contempt	8
4	religious nationalism	5
5	piety and religious nationalism	4
6	loyalty (towards a leader and government)	4
7	pride and religious nationalism	3
8	amusement	3
9	religious and political self preservation	2
10	joy	2
11	excitement	2
12	anger	2
13	surprise	1
14	self preservation	1
15	patriotism	1
16	national symbolism	1
17	hatred and contempt	1
18	disappointment	1
19		1

Table S4. Nearest neighbors in embedding space to "JaiShriRam" and "Kabir". From these lists, we included alternative spellings referencing JSR and Kabir in the construction of the explanatory variables in the empirical analysis.

Rank	Neighbor to #jaishriram	Similarity	Incl.	Reason
1	#jaihanuman	0.93	No	Other religious reference
2	#hindutva	0.93	No	Political movement
3	#jaishreeram	0.93	Yes	JSR with different spelling
4	#hindu	0.92	No	Hindu reference
5	#hindurashtra	0.91	No	Hindu nationalism
6	#ram	0.91	No	Reference to Rama, but not JSR
7	#hanumanji	0.91	No	Other religious reference
8	#जय_श्रीकृष्ण	0.91	No	Other religious reference (“Long live Shri Krishna”)
9	#जय_हिन्द_जय_भारत	0.90	No	Political reference (“Victory to India”)
10	#ramnavami	0.90	No	Religious festival (“Rama Navami”)
Rank	Neighbor to #जयश्रीराम	Similarity	Incl.	Reason
1	#जय_श्री_राम	0.81	Yes	JSR in Hindi (“Jai_Shri_Ram”)
2	#जय_श्रीराम	0.81	Yes	JSR in Hindi (“Jai_ShriRam”)
3	#जयश्रीराम	0.81	Yes	JSR in Hindi (“JaiShriRam”)
4	# !!जय	0.76	No	Different meaning (“Victory”)
5	#हर_हर_महादेव	0.75	No	Other religious reference (“Everywhere_Shiva”)
6	#आर्मी_कमांडो	0.74	No	Different meaning (“Army_Commando”)
7	#शुभ_प्रभात	0.74	No	Different meaning (“Good_morning”)
8	#जय____श्री___राम	0.74	Yes	JSR in Hindi (“Jai_____Shri_____Ram”)
9	#हर__हर__महादेव	0.74	No	Other religious reference (“Everywhere___Shiva”)
10	#शुभप्रभात	0.74	No	(“Good_morning”)
Rank	Neighbor to #kabir	Similarity	Incl.	Reason
1	#godkabir	0.97	Yes	Kabir reference
2	#kabira	0.97	Yes	Kabir reference
3	#god	0.97	No	General religious reference
4	#trueguru	0.96	No	Not clearly related to Kabir
5	#jannah	0.96	No	Islamic religious reference
6	#satguru	0.96	No	Ambiguous reference to either Kabir’s writing or Sikhism
7	#lord	0.96	No	General religious reference
8	#spirituality	0.95	No	General religious reference
9	#allahkabir	0.95	Yes	Kabir reference (specific to India)
10	#spiritual	0.95	No	General religious reference
Rank	Neighbor to #कबीर	Similarity	Incl.	Reason
1	#उदाह्यत	0.85	No	Seemingly unrelated meaning (“For example”)
2	#*कबीर	0.84	Yes	Kabir reference (“*Kabir”)
3	#इती	0.82	No	Seemingly unrelated meaning (“It is”)
4	#supremegodkabir	0.82	Yes	Kabir reference
5	#सत_भक्ति_संदेश	0.82	No	General religious reference (“true_devotion_message”)
6	#kabirisgod	0.81	Yes	Kabir reference
7	#संतरामपालजीमहाराज	0.80	No	Other religious reference to Sant Rampal (“Saint Rampal Ji Maharaj”)
8	#santkabir	0.80	Yes	Kabir reference
9	#सर्व	0.80	No	Seemingly unrelated meaning (“All”)
10	#624वां_कबीरसाहेब_प्रकटदिवस	0.79	No	624th anniversary of Kabir’s appearance

5. Results from the main analysis, robustness checks, and additional tests

Table S5. Results of the main models

	PB		LDV with week FE		TWFE		TWFE with TVC	
	JSR	Kabir	JSR	Kabir	JSR	Kabir	JSR	Kabir
Koos	0.114***	-0.138	0.181***	-0.484**	0.164***	-0.491**	0.164***	-0.489**
	(0.030)	(0.186)	(0.016)	(0.144)	(0.000)	(0.150)	(0.002)	(0.149)
Previous day's attacks (lagged outcome)			0.076**	0.075**				
			(0.021)	(0.021)				
Previous day's internet outages					0.001	(0.001)		
					(0.001)	(0.001)		
India-Pakistan relations (ICEWS)							0.029**	0.029***
							(0.008)	(0.006)
Intercept	0.010	0.019*						
	(0.006)	(0.008)						
Unit FE	No	No	No	No	Yes	Yes	Yes	Yes
Week FE	No	No	Yes	Yes	Yes	Yes	Yes	Yes
N	5300	5300	5300	5300	5300	5300	5300	5300
Adjusted R^2	0.000	0.000	0.013	0.013	0.038	0.038	0.038	0.038

Note: All Koo variables are normalized by the total number of Koo impressions. Standard errors are clustered by state and day. "PB" indicates the pooled bivariate model; "LDV" indicates the lagged dependent variable model with week fixed effects; "TWFE" stands for the two-way fixed effect model; and "TWFE with TVC" indicates the two-way fixed effects model with time-varying covaraites. *** $p < 0.001$; ** $p < 0.01$

Table S6. Results of the preferred model using the Koo predictors with larger temporal aggregations

Aggregation (number of days)	Koo type	Results of Koo predictor				
		Estimate	SE	p-value	N	Adjusted R^2
2	JSR	0.163	0.002	0.000	5290	0.03
2	Kabir	-0.489	0.149	0.010	5290	0.038
3	JSR	0.161	0.001	0.000	5280	0.038
3	Kabir	-0.505	0.157	0.010	5280	0.038
4	JSR	0.161	0.001	0.000	5270	0.037
4	Kabir	-0.477	0.143	0.009	5270	0.037
5	JSR	0.166	0.003	0.000	5260	0.037
5	Kabir	-0.477	0.143	0.009	5260	0.037
6	JSR	0.169	0.006	0.000	5250	0.037
6	Kabir	-0.471	0.140	0.008	5250	0.037
7	JSR	0.165	0.002	0.000	5240	0.036
7	Kabir	-0.469	0.139	0.008	5240	0.036

Note: The results presented in this table suggest that the main results are robust to using larger temporal aggregations for the lagged Koo predictors. We obtain these reported results by using the preferred model specification, two-way fixed effects with time-varying covariates, to regress DOTO incidents on the mean prevalence of JSR and Kabir posts over the preceding two to seven days. All Koo variables are normalized by the total number of Koo impressions. Standard errors are clustered by state and day.

Table S7. Results of the main models when using the late end date

		Results of Koo predictor				
	Koo type	Estimate	SE	p-value	N	Adjusted R^2
Pooled bivariate	JSR	0.021	0.019	0.281	8440	0.000
Pooled bivariate	Kabir	0.236	0.189	0.212	8440	0.000
Lagged outcome (LDV) with week FE	JSR	0.090	0.031	0.019	8440	0.012
Lagged outcome (LDV) with week FE	Kabir	-0.150	0.203	0.479	8440	0.012
Two-way fixed effects	JSR	0.085	0.028	0.015	8440	0.037
Two-way fixed effects	Kabir	-0.153	0.201	0.467	8440	0.037
Two-way fixed effects with time-varying covariates	JSR	0.085	0.028	0.015	8440	0.037
Two-way fixed effects with time-varying covariates	Kabir	-0.155	0.199	0.458	8440	0.037

Note: These results draw on a version of the data with a later end date, December 24, 2022, rather than the preferred end date, February 14, 2022. For explanation of the late date, and why the main analysis uses an earlier date, see the discussion of the Koo data in the main text, the explanation of Figure 2 in the main section, and Section A of this Supplementary Information document. In the models that produced these results, all Koo variables are normalized by the total number of Koo impressions. Standard errors are clustered by state and day. “FE” stands for “fixed effects”; “LDV” stands for “lagged dependent variable”.

Table S8. Results of the alternative models

		Results of Koo predictor				
	Koo type	Estimate	SE	p-value	N	Adjusted R^2
Two-way fixed effects with time-varying covariates and LDV	JSR	0.174	0.005	0.000	5300	0.039
Two-way fixed effects with time-varying covariates and LDV	Kabir	-0.485	0.155	0.012	5300	0.040
Two-way fixed effects with time-varying covariates and time-unit trend	JSR	0.173	0.011	0.000	5300	0.039
Two-way fixed effects with time-varying covariates and time-unit trend	Kabir	-0.485	0.155	0.012	5300	0.039
Linear probability model with two-way fixed effects and time-varying covariates	JSR	0.161	0.001	0.000	5300	0.037
Linear probability model with two-way fixed effects and time-varying covariates	Kabir	-0.496	0.145	0.008	5300	0.037
Two-way fixed effects with time-varying covariates, logit	JSR	0.161	0.001	0.000	5300	
Two-way fixed effects with time-varying covariates, logit	Kabir	-0.496	0.145	0.008	5300	
Two-way fixed effects with time-varying covariates, Poisson	JSR	8.906	0.127	0.000	2979	
Two-way fixed effects with time-varying covariates, Poisson	Kabir	-21.204	11.258	0.060	2979	

Note: To help assess the robustness of the main results, we fit five alternative inferential models, using both the JSR and Kabir predictors. This table reports the results obtained from the models. All Koo variables are normalized by the total number of Koo impressions. All models use the preferred specification, TWFE with time-varying covariates, except for the two robustness checks that add further covariates or interactions. The linear probability and logistic regressions use a binary version of the DOTO outcome variable. Standard errors clustered by state and day. “LDV” stands for “lagged dependent variable”.

Table S9. Results of additional tests

Test	Koo type	Results of Koo predictor				
		Estimate	SE	p-value	N	Adjusted R^2
Reverse direction: JSR Koo impressions regressed on DOTO events	JSR	-0.001	0.001	0.486	5300	0.709
Reverse direction: Kabir Koo impressions regressed on DOTO events	Kabir	-0.001	0.000	0.211	5300	0.389
Placebo treatment: DOTO events regressed on Cricket Koo impressions	Cricket	-1.969	1.716	0.281	5300	0.037

Note: To evaluate the soundness of our interpretation of the main results, we conduct two additional tests: a reverse direction test for both the JSR and Kabir predictors and a placebo treatment test. See the main text for explanations of these tests. This table reports the results of these tests. All models use the preferred specification, two-way fixed effects with time-varying covariates. All Koo variables are normalized by the total number of Koo impressions. Standard errors clustered by state and day.

Table S10. Results of the endorsement test

		Results of Koo predictor				
	Koo type	Estimate	SE	p-value	N	Adjusted R^2
Before the BJP's endorsement	JSR	0.179	0.179	0.000	1600	0.020
Before the BJP's endorsement	Kabir	-0.093	0.191	0.637	1600	0.019
After the BJP's endorsement	JSR	0.161	0.002	0.000	3700	0.039
After the BJP's endorsement	Kabir	-0.625	0.174	0.005	3700	0.040

Note: To further evaluate the soundness of our interpretation of the main results, we conduct an additional test that leverages the BJP endorsement of Koo on February 9, 2021. The logic of the test rests on the premise that the relationship between JSR or Kabir impressions and subsequent attacks should exist only after the endorsement, or when likely perpetrators are consuming the JSR and Kabir posts. See the main text for a detailed explanation of this endorsement test. This table reports the results of the test. All Koo variables are normalized by the total number of Koo impressions. All models use the preferred specification, TWFE with time-varying covariates. Standard errors clustered by state and day.

Table S11. Descriptive statistics for the panel data

Variable	Mean	Median	Interquartile Range	Minimum	Maximum	N
DOTO events	0.018	0	0	0	2	5310
DOTO events (binary)	0.018	0	0	0	1.000	5310
JSR impressions (normalized, with one day lag)	0.076	0.075	0.044	0.021	0.195	5300
Kabir impressions (normalized, with one day lag)	0.007	0.004	0.006	0	0.084	5300
Cricket impressions (normalized, with one day lag)	0	0	0	0	0.01	5300
ICEWS metric	0.003	0	0	0	4	5310
Internet outages (one day lag)	0.023	0	0	0	30	5300

Note: Descriptive statistics for all variables used in the panel data analysis.

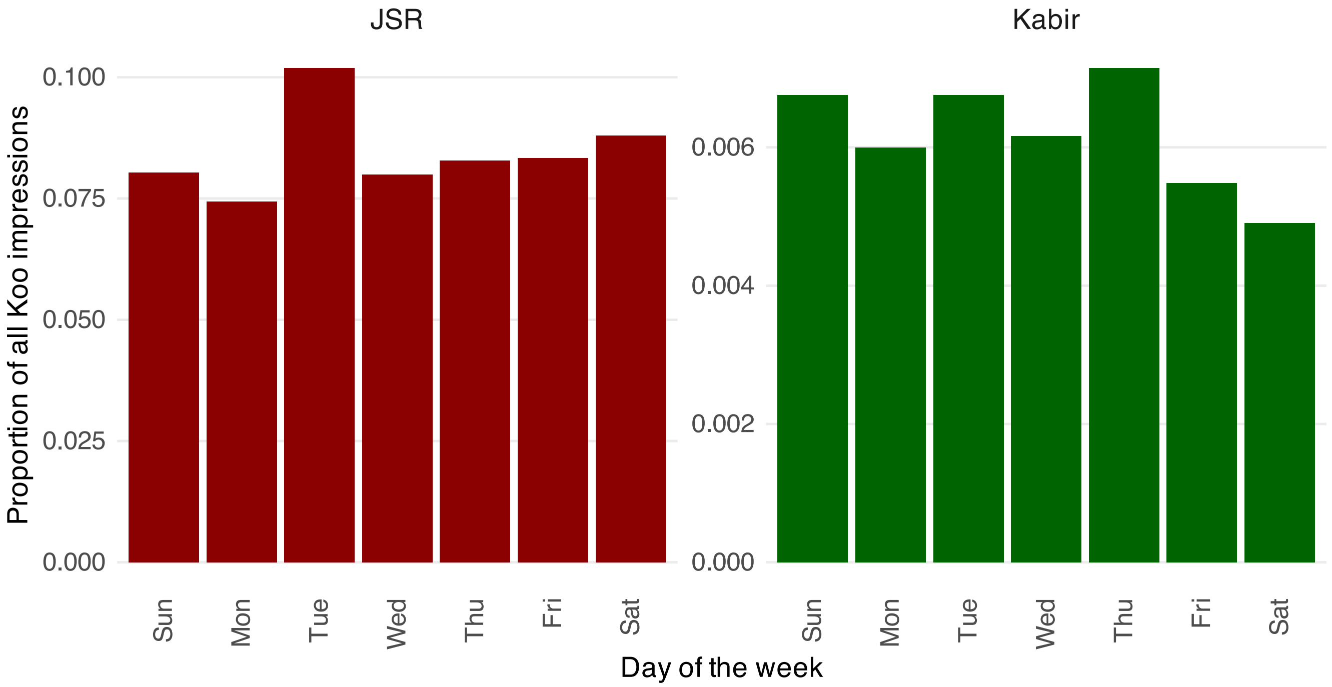

Fig. S1. The proportion of all Koo impressions which are labeled as JSR or Kabir impressions across days of the week during the study period. Take not of the different vertical axis scales.

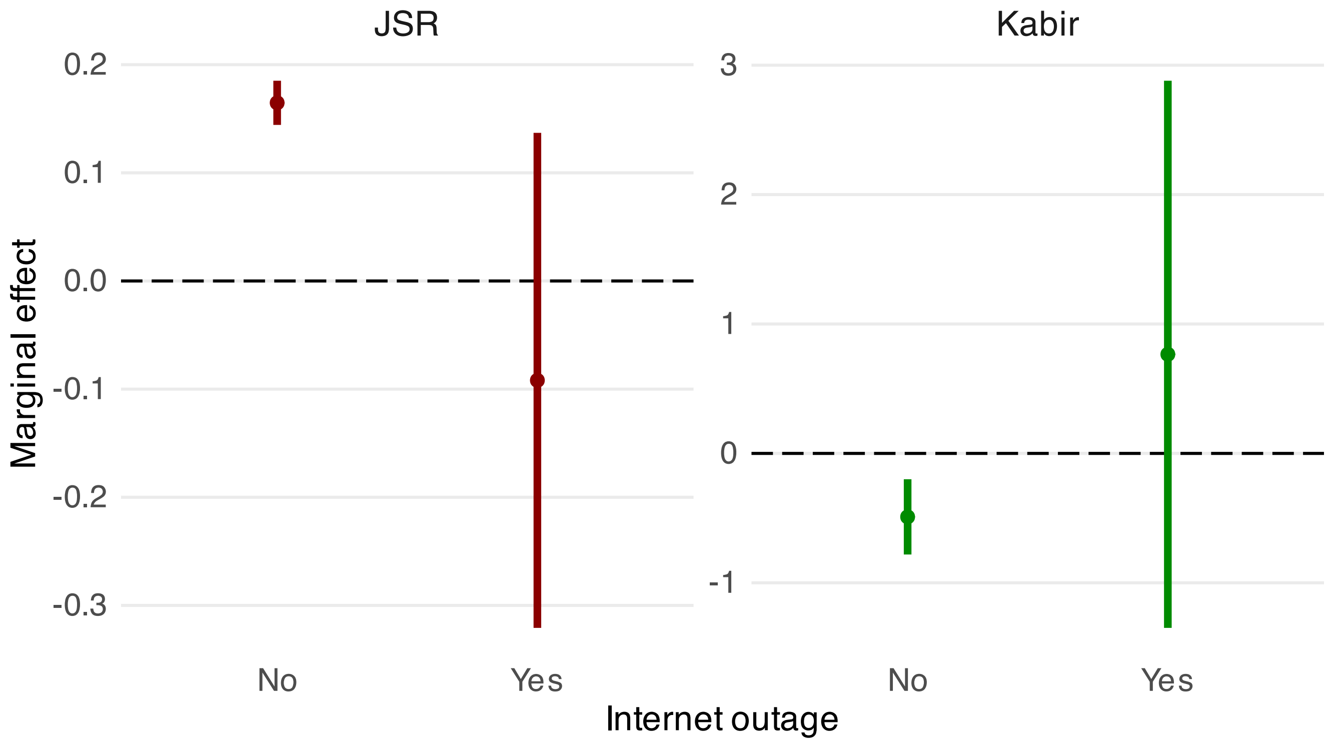

Fig. S2. The relationship between JSR and Kabir impressions and offline DOTO events is moderated by the occurrence of an Internet outage in a state. Bars denote 95% confidence intervals. Take note of the different vertical axis scales.

6. Discussion of alternative event data sources

The main analysis relies on a dataset obtained from a civil society organization in India, which focuses on minority rights. These "Documentation Of The Oppressed" (DOTO) data provides numerous advantages over more established event data collections, as it codes sources (*e.g.*, news articles, police reports, or civil society networks); perpetrators (to the extent that they are known); and, most importantly, the identities of victims. DOTO also offers a comprehensive coverage of event types, ranging from presumably organized lethal violence to spontaneous harassment. In the context of the Hindu-Muslim conflict in India, such coverage is needed, as a narrower focus might exclude important events.

In direct comparisons, the Global Terrorism Database (GTD) does not cover communal violence typically (2). The Social Conflict Analysis Database (SCAD) is focused on Africa and Latin America only (3). UCDP's GED only covers lethal events, involving organized actors rather than spontaneous crowds. As a consequence, it has relatively few observations for communal attacks (4). The ICEWS data (2), already in use in our models to measure international tensions with Pakistan, relies on automatically coded events from news sources. The communal attacks we focus on as a outcome variable are not systematically reported in international media. This leaves the Armed Conflict Location and Event Dataset (ACLED) (5) which does provide coverage for India.

Despite its wide coverage and many other advantages, ACLED has a critical limitation for our specific application: it is generally tacit on the identities of victims and their religious orientations. In addition, ACLED's otherwise very comprehensive list of *perpetrating* groups is also of little help because we are interested in low-level communal frictions and attacks. In India (and we suspect in comparable settings across the Global South), most small-scale attacks, such as attacks on a handful of religious or ethnic minorities, are not the official doing of an established paramilitary actor.

It would be possible to intentionally select among organizations and event types that are likely associated with some attacks, such as Cow protection groups, or the Rashtriya Swayamsevak Sangh (RSS). Still, such selections would presuppose involvement of specifics actors, and miss out on the possibility of spontaneous attacks. For these reasons, the only information ACLED can supply is whether or not a riot took place, without being able to pinpoint who attacked whom. As a result, using ACLED to construct our outcome variable would introduce significant measurement error. Nevertheless, we used ACLED and re-ran our main analysis to evaluate the robustness of our main results to using . As discuss in the following section (7), we obtain results that broadly align with the main findings (using DOTO).

7. Replication of the analysis with ACLED event data

To assess the robustness of our main results with regard to the data source for the outcome variable, we replicated the main analysis using the ACLED data collection. (See section 6 for a discussion of data sources). It is important to note that ACLED does not provide the same granularity of information as DOTO: rather than being able to focus on anti-Muslim and anti-Christian attacks specifically, we can only focus on general event types. In our case, these events are riots. Critically, because of the lack of information on victims, these riots can occur among or between any type of group, not only Hindus and religious minorities. We identify 2641 riots in India during our period of analysis; these are the observations of our outcome.

As seen in Figure S3, the replication of the main results when using ACLED broadly aligns with the results we obtain when using data from DOTO. The coefficient for JSR impressions remains significant at conventional levels for the three reasonable models (*i.e.*, the models with units and time

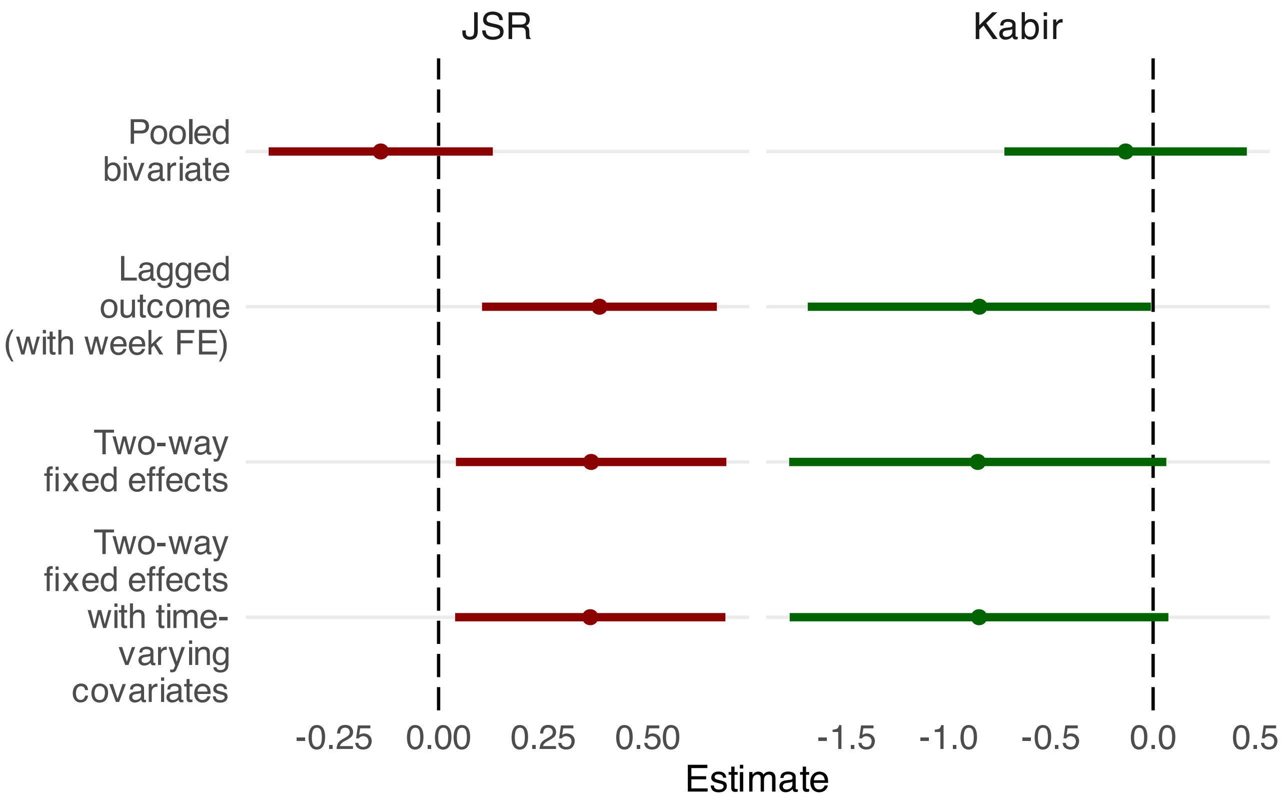

Fig. S3. Inferential results obtained when replicating the main analysis with the outcome measured using data from ACLED. Horizontal bars denote the 95% confidence intervals.

fixed effects and/or a lagged dependent variable and/or time-varying covariates). The coefficient for Kabir expressions is statistically significant when using the lagged dependent variable model but narrowly misses conventional levels of significance when using the two fixed effects models. However, for these two latter models, the direction of the association is consistent with the main results.

We interpret these findings as supportive of the main results and central conclusions. However, we emphasize that these findings are based on less than ideal data. The general riots reported in ACLED can occur between other religious groups, or between non-religious groups, such as supporters of different sports teams or political parties. This introduces a lot of variance into the analysis, and it is therefore not surprising that the replication with ACLED leaves us with higher levels of unexplained variance and less certainty in the estimates. Nevertheless, the broad agreement of the two distinct data sources helps to address concerns with drawing conclusions based on the DOTO data and, overall, increases our confidence in the main findings.

References

1. AK Singh, et al., What's kooking? characterizing india's emerging social network, koo in *Proceedings of the 2021 IEEE/ACM International Conference on Advances in Social Networks Analysis and Mining.* pp. 193–200 (2021).
2. G LaFree, L Dugan, Introducing the global terrorism database. *Terror. political violence* **19**, 181–204 (2007).
3. I Salehyan, et al., Social conflict in africa: A new database. *Int. Interactions* **38**, 503–511 (2012).
4. R Sundberg, E Melander, Introducing the ucdp georeferenced event dataset. *J. peace research* **50**, 523–532 (2013).
5. C Raleigh, A Linke, H Hegre, J Karlsen, Introducing acled: An armed conflict location and event dataset. *J. peace research* **47**, 651–660 (2010).
